# New Spiral γ-Lactone Enantiomers from the Plant Endophytic Fungus *Pestalotiopsis foedan*

**DOI:** 10.3390/molecules18022236

**Published:** 2013-02-11

**Authors:** Xiao-Long Yang, Zhuang-Zhuang Li

**Affiliations:** College of Pharmaceutical Science, Hebei University, Baoding 071002, Hebei, China

**Keywords:** plant endophytic fungus, *Pestalotiopsis foedan*, foedanolide, cytotoxic activity

## Abstract

(−)-(4*S*, 8*S*)-Foedanolide (**1a**) and (+)-(4*R*, 8*R*)-foedanolide (**1b**), a pair of new spiro-γ-lactone enantiomers, were isolated from the fermentation broth of the plant endophytic fungus *Pestalotiopsis foedan* by HPLC using a chiral column, achieving over 7% ee. Their structures and absolute configurations were determined on the basis of extensive analysis of NMR spectra combined with computational methods via calculation of the electronic circular dichroism (ECD) and optical rotation (OR). Compounds **1a** and **1b** showed moderate activities against HeLa, A-549, U-251, HepG2 and MCF-7 tumor cell lines.

## 1. Introduction

Endophytic fungi, as one of the most promising resource for natural product discovery, live inside the normal tissues of host plants without causing apparent disease symptoms [1]. The study of natural products from plants and their endophytes has shown that endophytes have been found to produce a significant number of interesting novel and bioactive metabolites [2]. For example, *Taxomyces andreanae*, the first report endophytic fungus colonizing the inner bark of Pacific yew *Taxus brevifolia*, is capable of producing taxol and its analogue baccatin III when grown in semi-synthetic medium [3]. One of the most commonly found endophytes is *Pestalotiopsis* genus [4]. Since discovery of the anticancer agent taxol from an endophytic fungal strain *Pestalotiopsis microspora* [5], interest in searching for bioactive compounds from this fungal genus has increased considerably. Previous chemical studies of some species of this genus have afforded a variety of bioactive metabolites [2,6,7,8,9].

**Figure 1:**
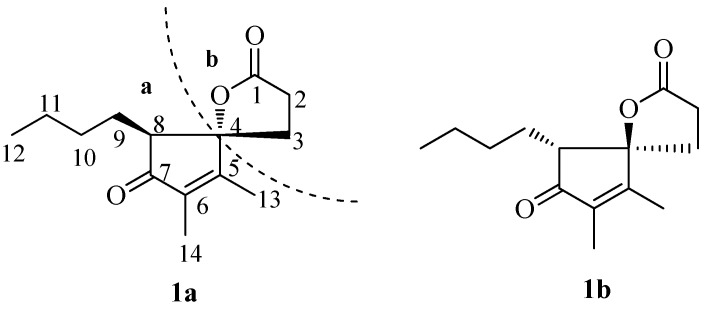
The structures of compounds **1a** and **1b**.

During our continuing research for naturally occurring bioactive secondary metabolites from this genus, the present study was undertaken to investigate the chemical constituents of *Pestalotiopsis foedan* isolated from the branch of *Bruguiera sexangula*, and have led to the isolation of a pair of new spiro-γ-lactone enantiomers **1a** and **1b** (Figure 1). Details of the isolation, structure elucidation and cytotoxic evaluation of **1a** and **1b** are reported herein.

## 2. Results and Discussion

Compound **1** was obtained as a white powder. Its molecular formula was determined to be C_14_H_20_O_3_ by high-resolution atmospheric pressure chemical ionization mass spectrometry (HR-APCI-MS) ([M+H]^+^, found 237.1501, calc. 237.1485), corresponding to five degrees of unsaturation. The IR spectral data of **1** showed the presence of one α,β-unsaturated ketone group (1780 cm^−1^) and one ester carbonyl group (1712 cm^−1^). Analysis of the ^1^H- and ^13^C-NMR spectral data (Table 1) revealed that **1** contained one α,β-unsaturated ketone carbonyl group, one ester carbonyl group, two olefinic quaternary carbons, three methyl groups, five methylene groups, one methine group, and one oxygenated sp^3^ quaternary carbon. The connectivity of the protons and carbons was established by the HSQC data. Two olefinic quaternary carbons could be assigned to one olefin, together with the two carbonyls accounted for three of the five required degrees of unsaturation. The remaining two degrees of unsaturation had to be accounted for two rings.

**Table 1:** ^1^H- (600 MHz) and ^13^C-NMR (150 MHz) data for **1** in CDCl_3._

No.	*δ_H_*	*δ_C_*	No.	*δ_H_*	*δ_C_*
1		176.0 (s)	8	2.67 (t, 6.6)	55.4 (d)
2	2.76 (t, 7.2)	29.4 (t)	9	1.85 (m), 1.38 (m)	25.4 (t)
3	2.30 (m), 2.16 (m)	26.1 (t)	10	1.65 (m), 1.38 (m)	30.1 (t)
4		92.1 (s)	11	1.38 (m)	22.9 (t)
5		163.9 (s)	12	0.93 (t, 7.2)	13.9 (q)
6		138.0 (s)	13	2.01 (s)	10.9 (q)
7		203.8 (s)	14	1.76 (s)	8.2 (q)

**Figure 2:**
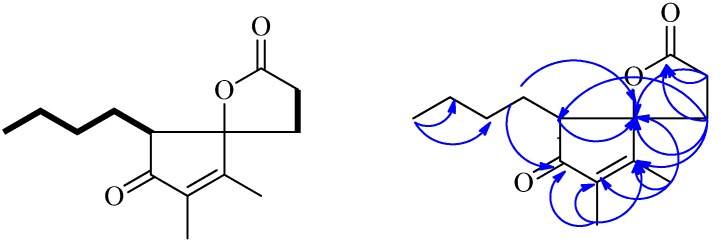
The ^1^H, ^1^H-COSY and selected HMBC correlations of **1**.

Cross-peaks between H-2 and H-3, H-8 and H-9/H-10/H-11/H_3_-12 were observed in the ^1^H, ^1^H-COSY spectrum (Figure 2). It allowed establishment of two H-atom systems, one is C-2 through C-3, and the other is C-8 through C-9, C-10, C-11 to C-12. HMBC correlations (Figure 2) of H-8 with C-4, C-7 and C-9, H-9 with C-4, C-7 and C-8, H-12 with C-10 and C-11, H_3_-13 with C-4, C-5 and C-6, and of H_3_-14 with C-5, C-6 and C-7, indicated that **1** contains the cyclopentenolone moiety with the *n*-butyl chain attached to C-8 position. Finally, fragment **a** was established (Figure 1). The γ-lactone moiety (fragment **b**) was established by analysis of the remaining HMBC (Figure 2) correlations of H-2 with C-1, C-3 and C-4, and of H-3 with C-1, C-2 and C-4, combined with the only remaining one degree of unsaturation which had to be accounted for one ring. In light of the evidences mentioned above, the planar structure of **1** was established (Figure 1).

Further interpretation of NOESYspectrum revealed that no NOE interactions were observed between H-3 and H-8, H-9. Thus, compound **1** may be either a *cis* or *trans* structure.

The recorded optical rotation (OR) for **1** was −8 (*c* = 0.25, MeOH), and the observed CD spectrum has a negative Cotton effect at 221 nm with a Δε value of 2.0. This is unexpected for a spiro compound due to its quite small optical rotation value, and we predicted that **1** might be isolated as a partially racemic mixture with low ee. Thus, compound **1** was finally separated by preparative HPLC on a Chiralcel OB-H phase to yield (−)-(4*S*,8*S*)-foedanolide (**1a**) and (+)-(4*R*,8*R*)-foedanolide (**1b**), revealing an ee of 7% for the unresolved mixture based on the integration value of HPLC peak areas. The major enantiomer **1a** has a OR value of −101.0 (*c* = 0.11, MeOH), and its CD spectrum has a Δε value of 21.5 at 221 nm.

**Figure 3:**
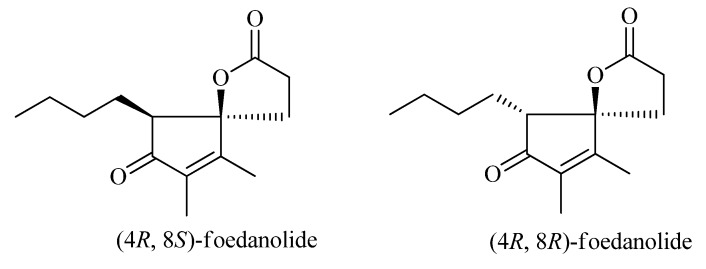
The structures of the two diastereomers, *cis* (4*R*,8*S*) and *trans* (4*R*,8*R*).

We tried to crystallize of **1a** and **1b** in different solvents, and unfortunately failed to obtain crystals. Finally, the absolute configurations of enantiomers were determined by computational methods [10,11,12,13,14]. Theoretically, compound **1** has four isomers. To reduce computational time, two diastereomers, *cis* (4*R*,8*S*) and *trans* (4*R*,8*R*) were selected for further computations (Figure 3).

**Figure 4:**
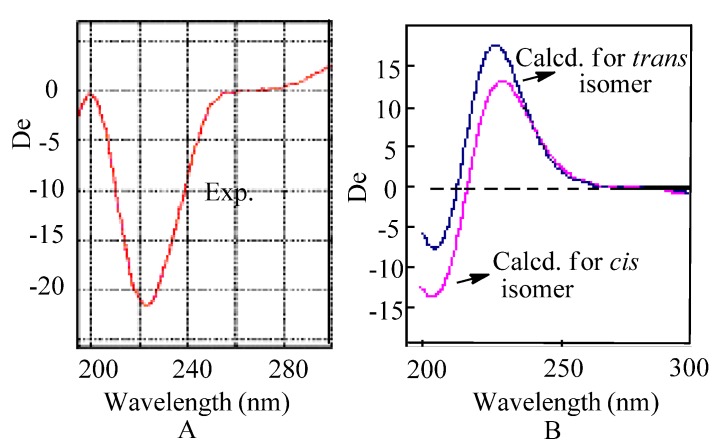
(**A**) Experimental CD; (**B**) Computed CD for *cis* and *trans* isomers.

To assign the configuration, electronic CD computations were performed at the B3LYP/6-311++G(2d,2p)//B3LYP/6-311++G(2d,p) level for the (4*R*,8*S*) and (4*R*,8*R*) isomers. It’s unexpected there was no obvious differences between their CD (Figure 4) [14]. However, from the CD spectra, it can be concluded that the configuration must be one of the (4*S*,8*S*) and (4*S*,8*R*). Finally, optical rotations (OR) for the *cis* and *trans* isomers were calculated at the B3LYP/6-311++G(2d,p)//B3LYP/ 6-31+G(d) level [15]. The computed OR for *trans* isomer (4*R*,8*R*) is +132.9 and for *cis* isomer (4*R*,8*S*) is +5.6. The experimental OR values of **1a** and **1b** are −101 (*c* = 0.11, MeOH), +103 (*c* = 0.15, MeOH), respectively. Therefore, the absolute configuration of **1b** was determined to be 4*R*,8*R*. Compound **1a** was also subjected to OR computation, it was found that it’s OR value is −132.9. Combing with the experimental OR value, the absolute configuration of the major enantiomer **1a** was finally determined to be 4*S*,8*S*.

Compounds **1a** and **1b** were tested for their cytotoxicity against a small panel of human tumor cell lines including HeLa, A-549, U-251, HepG2 and MCF-7 (Table 2). Both exhibited inhibitory activities against HeLa, HepG2 and MCF-7, and the cytotoxic activities of compound **1b** were all higher than compound **1a**. Importantly, compound **1b** showed significant activity against HeLa tumor cell line with IC_50_ value of 5.4 µg mL^−1^. Compound **1b** also exhibited inhibitory activities against A-549 and U-251 tumor cell lines with IC_50_ value of 67.9 and 53.0 µg mL^−1^, whereas compound **1a** was inactive against A-549 and U-251 cell lines at the same concentrations (IC_50_ < 100 µg/mL). It suggested that the stereochemistry of enantiomers could affect their cytotoxicity. Actually, this phenomenon was already reported for other enantiomers. For example, a popular herbicide, metolachlor, its (*S*)-metolachlor exhibited highly effective toward grasses, while (*R*)-enantiomer is inactive [16].

**Table 2:** Antitumor effects of (−)-foedanolide (**1a**) and (+)-foedanolide (**1b**) (IC_50_, µg/mL).

Compound	HeLa	A549	U251	HepG2	MCF-7
(-)-foedanolide (**1a**)	15.8	296.0	159.0	22.8	70.2
(+)-foedanolide (**1b**)	5.4	67.9	53.0	19.0	20.8
DPP(positive control)	4.5	8.6	8.5	0.7	4.3

## 3. Experimental

### 3.1. General

Optical rotations: JASCO P-1020 spectropolarimeter (JASCO International Co., Ltd., Easton, MD, USA). UV spectra: UV-210 spectrometer (Tokyo Rikakikai Co. Ltd, Tokyo, Japan), λ_max_ (log ε) in nm. CD spectra: JASCO J-815 spectropolarimeter (JASCO International Co., Ltd.). IR spectra: Perkin-Elmer 577 spectrometer (PerkinElmer Corporation, Waltham, MA, USA), KBr pellets; in cm^−1^. NMR spectra: Bruker AM-600 spectrometer (Bruker Corporation, Munich, Germany), *δ* in ppm, *J* in Hz, Me_4_Si as internal standard. FT-MS spectra: Bruker apex-ultra 7.0 T spectrometer (Bruker Corporation) in *m/z*. Column chromatography (CC): silica gel (200~300 mesh, Yantai Zhi Fu Chemical Co. Ltd., Yantai, China), TLC: silica gel GF_254_ plates (Yantai Zhi Fu Chemical Co. Ltd.) and Sephadex LH-20 gel (25~100 μm, GE Healthcare Co. Ltd., Uppsala, Sweden).

### 3.2. Fungal Material and Cultivation Conditions

*Pestalotiopsis foedan* was isolated from the branches of *Bruguiera sexangula* in Hainan, China, in April, 2008, identified by Prof. Jing-Ze Zhang, and assigned the accession number L444. The fungal strain was cultured on slants of potato dextrose agar (PDA) at 28 °C for 7 days, and then inoculated into 500 mL Erlenmeyer flask containing 100 mL of PDB (20.0 g of glucose, 200.0 g of potato (peeled), 3.0 g of KH_2_PO_4_, 1.5 g of MgSO_4_, 0.1 g of citric acid, and 10.0 mg of thiamin hydrochloride, in 1 liter of deionized H_2_O). The final pH of the media was adjusted to 6.5 before sterilization. After 7 days of incubation at 28 °C on rotary shakers at 150 rpm, 25 mL of culture liquid were transferred as seed into each 1,000 mL Erlenmeyer flask containing 250 mL of PDB and fermentation was carried out on a shaker for 30 days.

### 3.3. Extraction and Isolation

The culture broth (40 L) was extracted three times with ethyl acetate (40 L/each time, 12 h). Evaporation of the solvent *in vacuo* gave a brown oily residue (40.0 g), which was subjected to column chromatography (silica gel) with gradient elution systems of petroleum ether/acetone (from 100:0 to 0:100). The fraction (6.9 g) eluted with 80% petroleum ether was further purified by repeated CC (petroleum ether/acetone, 40:1) and Sephadex LH-20 chromatography (acetone) to afford compound **1** (4.0 mg). Compound **1** was dectected as one spot on TLC by heating silica gel plates sprayed with 10% H_2_SO_4_ in ethanol, and which also exhibited one peak monitored by HPLC. The recorded optical rotation (OR) for **1** was −8 (*c* 0.25 MeOH), the observed CD had a negative Cotton effect at 221 nm with only a Δε values of 2.0. This is unexpected for a spiral compound. It is doubt that another enantiomer mixed inside. Thus, compound **1** was further isolated by HPLC on a chiralrel OB-H phase (80% n-hexane in 20% isopropanol over 30 min, 1 mL/min, 225 nm, 25 °C), which led to the isolation of (−)-(4*S*,8*S*)-foedanolide (**1a**) (1.7 mg, *t_R_* = 13.5 min) and (+)-(4*R*,8*R*)-foedanolide (**1b**) (1.5 mg, *t_R_* = 17.6 min).

*Foedanolide* (**1**): Isolated as white powder; [*α*]_D_^19.7^ = −8° (*c* = 0.25, MeOH). UV (CHCl_3_) λ_max_ (lg *ε*): 227 (5.10), 373 (3.36) nm. IR (KBr) v_max_: 1780 (C=O), 1712 (C=O), 1660 (C=C) cm^−1^. ^1^H- and ^13^C-NMR: see Table 1. Positive ion HR-APCI-MS [M+H]^+^*m/z* 237.1501 (calcd for C_14_H_21_O_3_, 237.1485).

### 3.4. Cytotoxicity Assay

Cytotoxicity activity was evaluated against HeLa, A-549, U-251, HepG2 and MCF-7 cells by the MTT method [17]. All cell lines were grown in RPMI-1640 medium (GIBCO) supplemented with 10% heat-inactivated bovine serum, 2 nM L-glutamine, 10^5^ IU/L penicillin, 100 mg/L streptomycin and 10 mM HEPES, pH 7.4. Cells were kept at 37 °C in a humidified 5% CO_2_ incubator. An aliquot (180 μL) of these cell suspensions at a density of 1,500 cell mL^−1^ was pipetted into 96-well microtiter plates. Subsequently, 180 μL of sample (in DMSO) at different concentrations was added to each well and incubated for 72 h at the above conditions in a CO_2_-incubator. MTT solution (20 µL of 5 mg/L in RPMI-1640 medium) was added to each well and further incubated for 4 h at 37 °C. After addition of 100 µL DMSO and incubation for 1h, the cells were lysed to liberate the formed formazan crystals. The optical density (OD) was read on a Multiscan plate reader at a wavelength of 570 nm. DMSO control well, in which sample was absent, was included in the experiment in order to eliminate the influence of DMSO. The inhibitory rate of cell proliferation was calculated by the following formula:

Growth inhibition (%) = [OD_control_ − OD_treated_/OD_control_] × 100% (1)

The cytotoxicity of samples on tumor cells was expressed as IC_50_ values and calculated by LOGIT method.

## 4. Conclusions

A pair of new spiro-γ-lactone enantiomers was isolated from the fermentation broth of the plant endophytic fungus *Pestalotiopsis foedan* by HPLC using a chiral column. Their absolute configurations were mainly determined by computational methods *via* calculation of the electronic circular dichroism (ECD) and optical rotation (OR). Preliminary biological assay of **1a** and **1b** showed that both compounds exhibited moderate activities against HeLa, A-549, U-251, HepG2 and MCF-7 tumor cell lines.

## Supporting Information

Supplementary materials can be accessed at: http://www.mdpi.com/1420-3049/18/2/2236/s1.
