# Supplementary material for: New Spiral γ-Lactone Enantiomers from the Plant Endophytic Fungus Pestalotiopsis foedan
Source: Molecules. 2013 Feb 11;18(2):2236–42. doi: 10.3390/molecules18022236 (PMC6269859; doi:10.3390/molecules18022236)

Supporting Information

Computation Section

1. Computation of optical rotation for two diastereomers, *cis* (4*R*, 8*S*) and *trans* (4*R*, 8*R*)

Table 1. The computed optical data for *trans* (4*R*, 8*R*) – enantiomer.

entry	OR	Energy
1	181.71	−771.32079
2	108.10	−771.3192912
3	222.60	−771.3194106
4	142.08	−771.3197426
5	143.53	−771.3179848
6	155.10	−771.3194675
7	64.03	−771.3200188
8	177.30	−771.3177407
9	74.41	−771.3181749
10	189.87	−771.3183581
11	116.45	−771.3183658
12	74.62	−771.318175
13	55.23	−771.3200096
14	89.14	−771.3187781
Sum of OR	+132.9	

Table 2. The computed optical data for *cis* (4*R*, 8*S*) – enantiomer.

Entry	OR	Energy
1	−23.51	−771.3195611
2	−131.99	−771.3207403
3	144.67	−771.3205974
4	−76.05	−771.3197859
5	−73.70	−771.3183182
6	−57.19	−771.3202697
7	−164.70	−771.3194685
8	36.09	−771.318334
9	103.80	−771.3192806
10	44.95	−771.318179
11	161.07	−771.3187903
12	60.47	−771.3194445
13	62.63	−771.3184976
14	51.85	−771.3191076
15	79.57	−771.3209678
16	27.66	−771.3187731
17	44.92	−771.3196886
18	85.33	−771.3191618
19	−9.39	−771.3194409
Sum of OR	+5.6	

Figure S1. ¹H-NMR spectrum of **1** (600 MHz, $CDCl_3$).

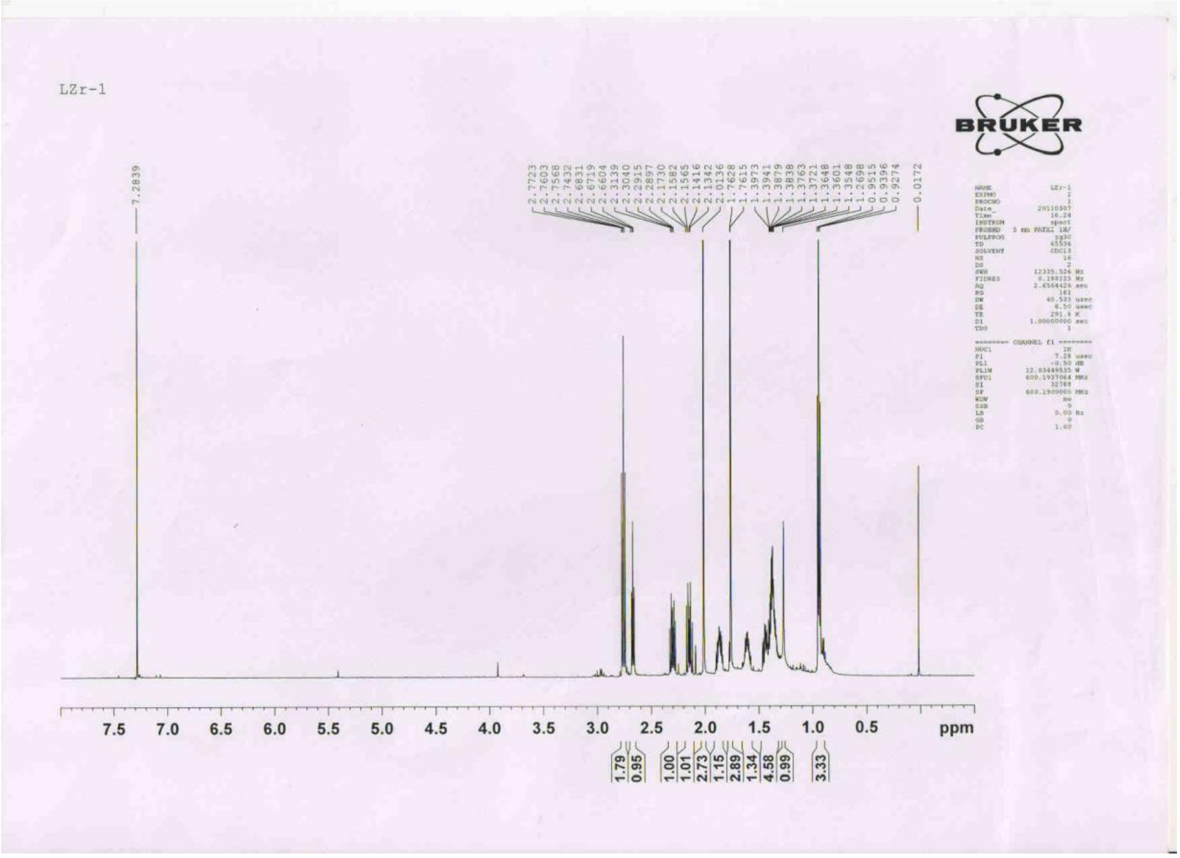

Figure S2. ¹³C-NMR spectrum of **1** (150 MHz, $CDCl_3$).

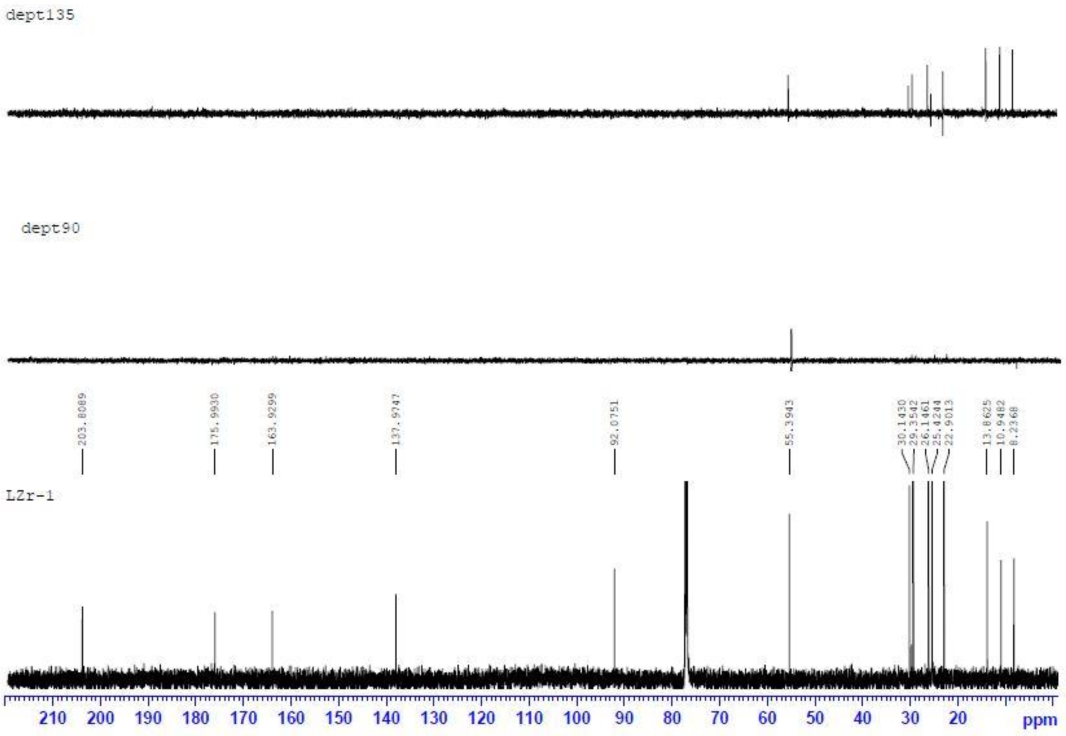

Figure S3. HSQC spectrum of **1** ($CDCl_3$).

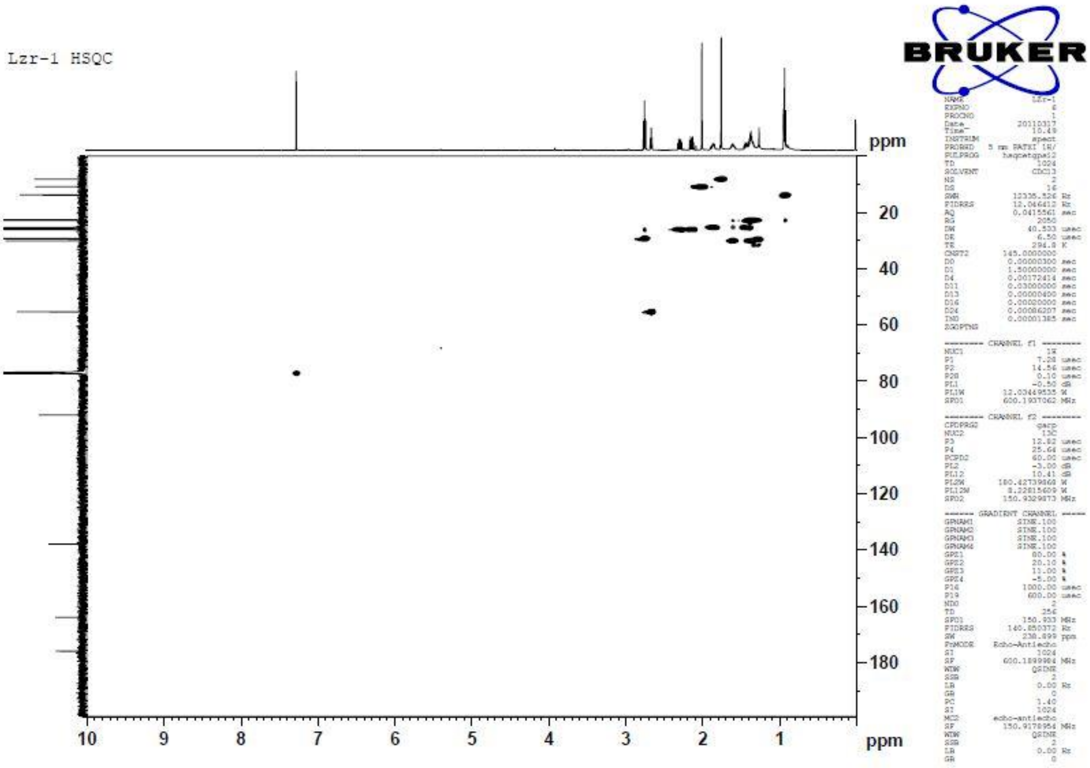

Figure S4. HMBC spectrum of **1** ($CDCl_3$).

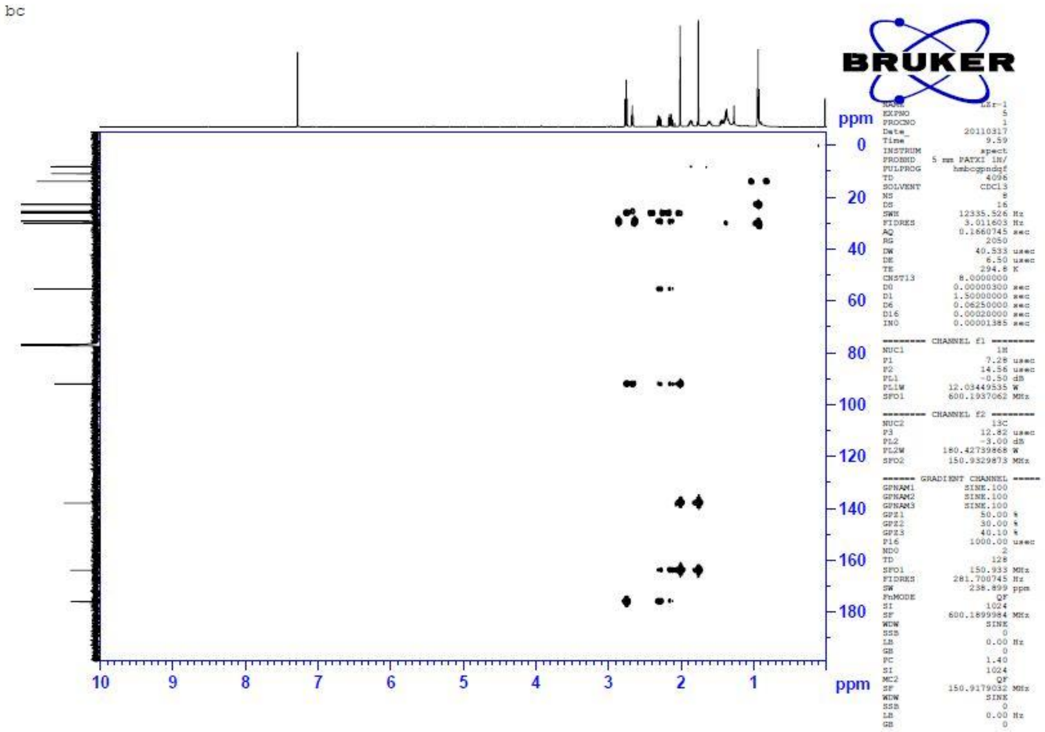

Figure S5. $^{1}H,^{1}H$-COSY spectrum of **1** ($CDCl_3$).

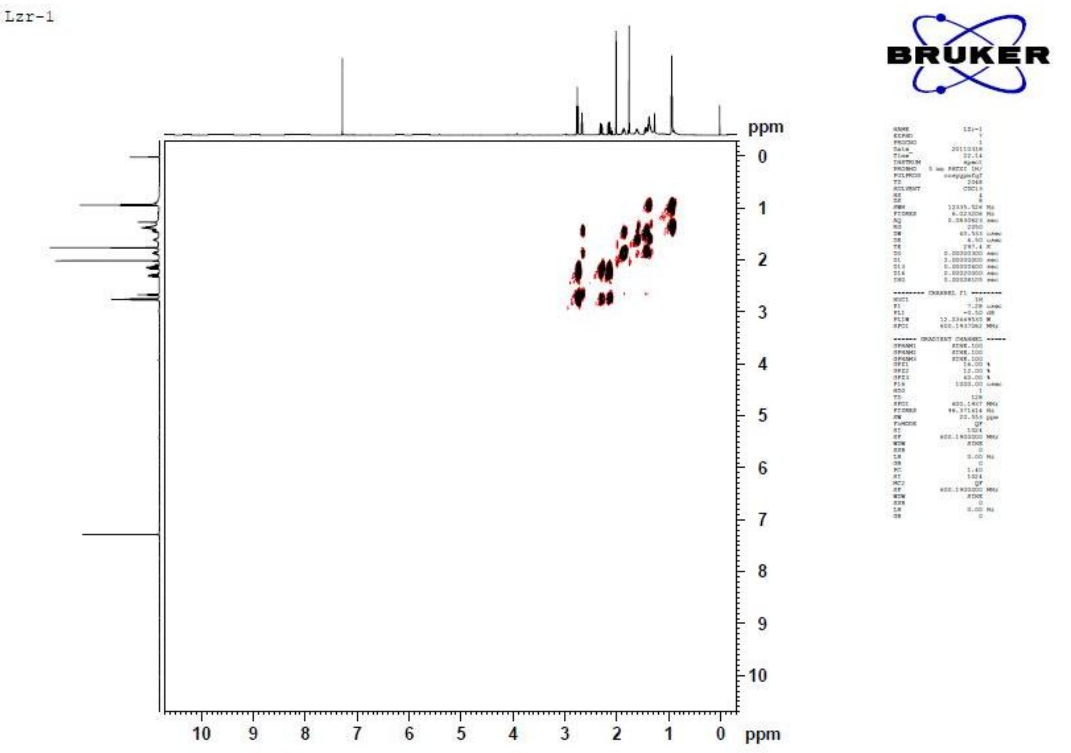

Figure S6. NOESY spectrum of **1** ($CDCl_3$).

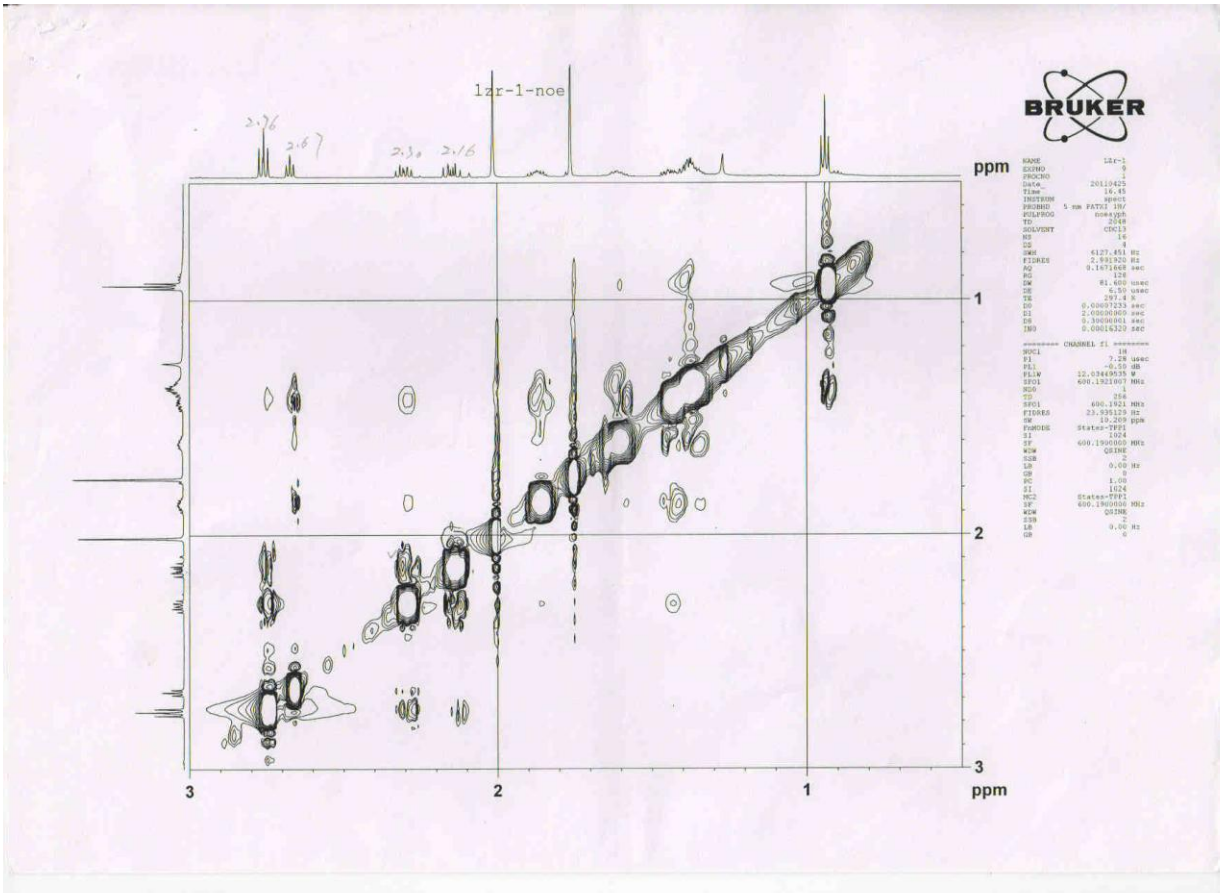

Figure S7. HR-APCI-MS spectrum of **1** ($CDCl_3$).

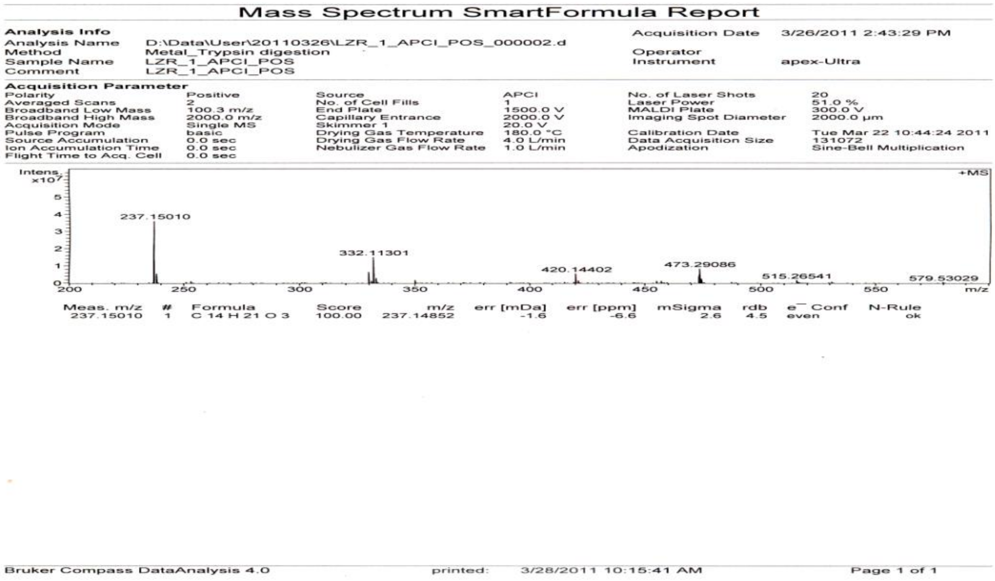

Figure S8. IR spectrum of **1**.

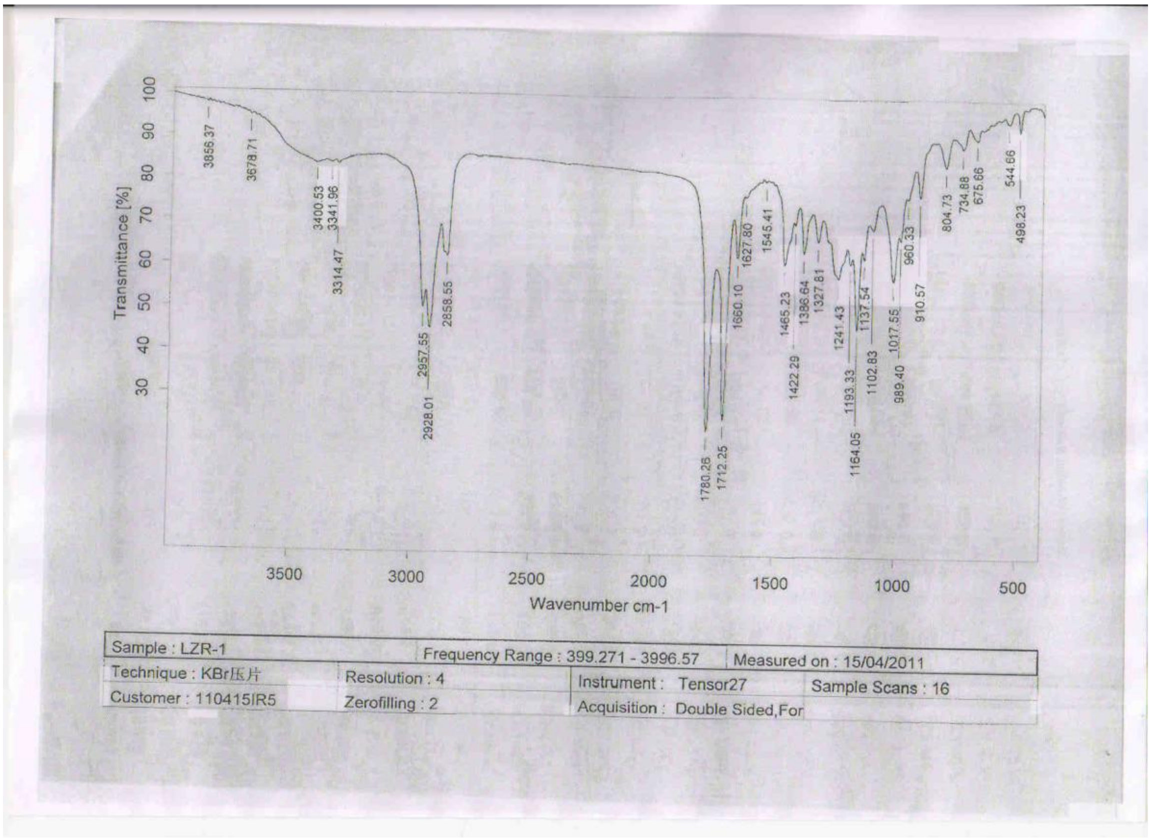

Figure S9. UV spectrum of **1**.

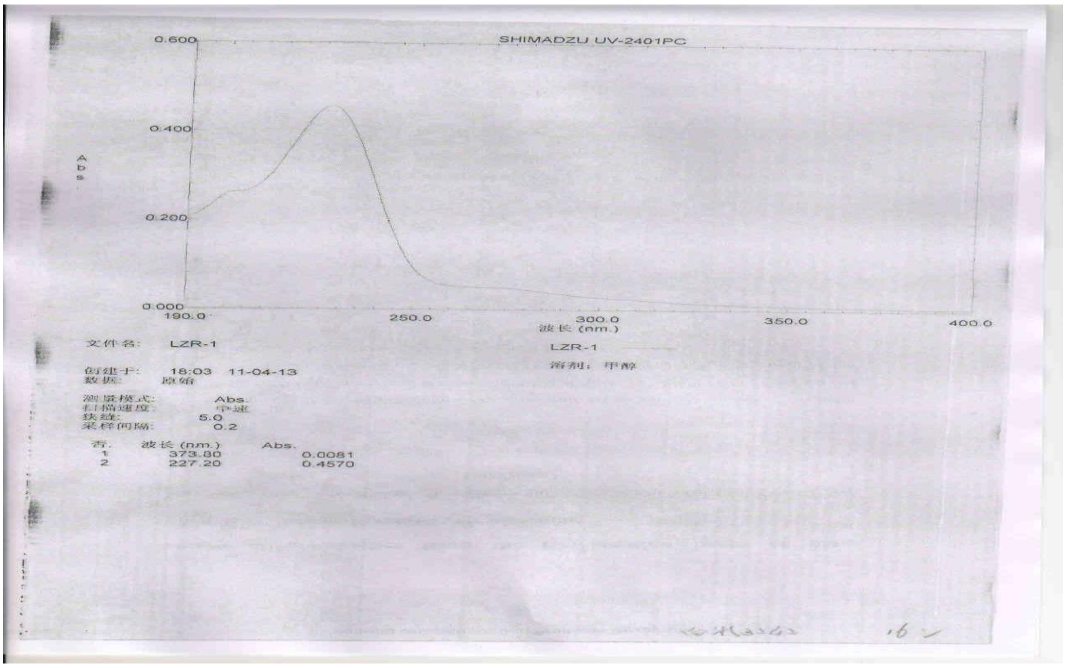

Figure S10. CD spectrum of (−)-foedanolide (**1a**).

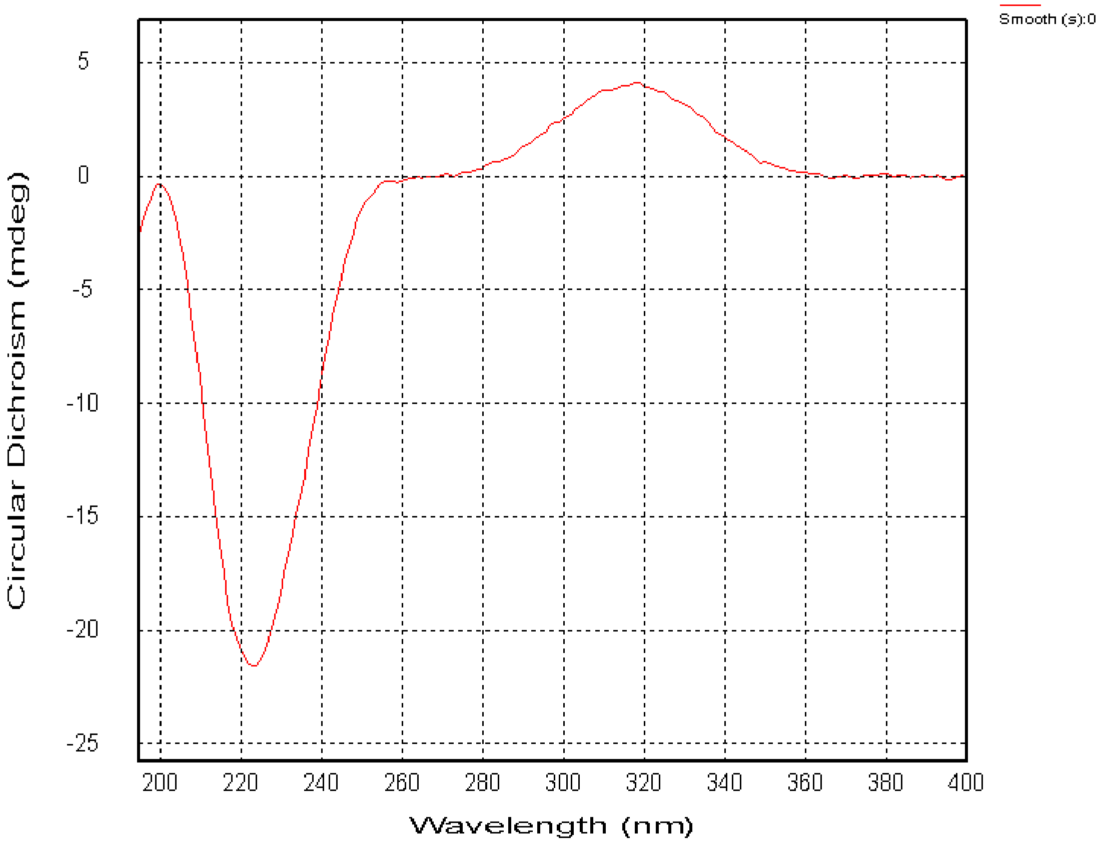

Supplement: Supplementary file 1 [file molecules-18-02236-s001.pdf]
